# Superoptimal Rate of Convergence in Nonparametric Estimation for Functional Valued Processes

**DOI:** 10.1155/2014/264217

**Published:** 2014-09-16

**Authors:** Christophe Chesneau, Bertrand Maillot

**Affiliations:** LMNO, CNRS, Université de Caen, Campus II, Science 3, 14032 Caen, France

## Abstract

We consider the nonparametric estimation of the generalised regression function for continuous time processes with irregular paths when the regressor takes values in a semimetric space. We establish the mean-square convergence of our estimator with the same superoptimal rate as when the regressor is real valued.

## 1. Introduction

Since the pioneer works of [1, 2], the nonparametric estimation of the regression function has been very widely studied for real and vectorial regressors (see, e.g., [3–8]) and, more recently, the case when the regressor takes values in a semimetric space of infinite dimension has been addressed. Interest in this type of explanatory variables has increased quickly since the foundational work of Ramsay and Silverman (1997), who proposed efficient methods for linear modelling (see [9] for a reissue of this work or [10, 11] for other developments on this topic). Later, fully nonparametric methods have been proposed (e.g., [12–15]) but the increased generality comes at a price in terms of convergence rate: in the regression estimation framework, it is well known that the efficiency of a nonparametric estimator decreases quickly when the dimension of the regressor grows. This problem, known as the “curse of dimensionality,” is due to the sparsity of data in high dimensional spaces. However, when studying continuous time processes with irregular paths, it has been shown in [16] that even when the regressor is *ℝ* ^*d*^-valued, we can estimate the regression function with the parametric rate of convergence $𝒪\left(1/\sqrt{T}\right)$. This kind of superoptimal rate of convergence for nonparametric estimators is always obtained under hypotheses on the joint probability density functions of the process which are very similar to those introduced by [17]. Since there is no equivalent of the Lebesgue measure on an infinite-dimensional Hilbert space, the definition of a density is less natural in the infinite-dimensional framework and the classical techniques cannot be applied. Under hypotheses about probabilities of small balls, we show that we can reach superoptimal rates of convergence for nonparametric estimation of the regression function when the regressor takes values in an infinite-dimensional space.

Notations and assumptions are presented in Section 2.  Section 3 introduces our estimator and the main result. We comment on hypotheses and results and give some examples of processes fulfilling our hypotheses in Section 4. A numerical study can be found in Section 5. The proofs are postponed to Section 6.

## 2. Problem and Assumptions

Let {*X*_*t*_, *Y*_*t*_}_*t*∈[0,*∞*)_ be a measurable continuous time process defined on a probability space (*Ω*, *ℱ*, *P*) and observed for *t* ∈ [0, *T*], where *Y*_*t*_ is real valued and *X*_*t*_ takes values in a semimetric vectorial space *ℋ* equipped with the semimetric *d*(·, ·). We suppose that the law of (*X*_*t*_, *Y*_*t*_) does not depend on *t* and that there exists a regular version of the conditional probability distribution of *Y*_*t*_, given *X*_*t*_ (see [18–20] for conditions giving the existence of the conditional probability). Throughout this paper, *𝒞* denotes a compact set of *ℋ*. Let Ψ be a real valued Borel function defined on *ℝ* and consider the generalized regression function

(1) $$\begin{array}{c} r\left(x\right)∶=E\left(\Psi \left(Y_{0}\right)\;|\;X_{0}=x\right),\;x\in C. \end{array}$$

We aim to estimate *r* from {*X*_*t*_, *Y*_*t*_}_*t*∈[0,*T*]_.

We gather hereafter the assumptions that are needed to establish our result.

(H1) For any *x* ∈ *ℋ* and any *h* > 0, set *ℬ*(*x*, *h*)∶ = {*y* ∈ *𝒞*; *d*(*y*, *x*) ≤ *h*}. There exist three constants (*c*_1_, *C*, *η*)∈(0, *∞*)^2^ × ]0,1] such that, for any *x* ∈ *𝒞* and any (*u*, *v*) ∈ *ℬ*(*x*, *c*_1_)^2^, we have
  (2) $$\begin{array}{c} \left|r\left(u\right)-r\left(v\right)\right|\leq Cd{\left(u,v\right)}^{\eta }. \end{array}$$
(H2) There exist
  (i) a function *ϕ* and three constants (*β*_1_, *β*_2_, *c*_2_) ∈ [0,*∞*)^3^ such that, for any *x* ∈ *𝒞* and any *h* ∈ (0, *c*_2_], we have
    (3) $$\begin{array}{c} 0<\beta_{1}\phi \left(h\right)\leq P\left(X_{0}\in B\left(x,h\right)\right)\leq \beta_{2}\phi \left(h\right), \end{array}$$
  (ii) a constant *c*_3_ > 0 and a function *g*_0_ integrable on (0, *∞*) such that, for any *x* ∈ *𝒞*, any *s* > *t* ≥ 0, and any *h* ∈ (0, *c*_3_], we have
    (4) |P((Xt,Xs)∈B(x,h)2)−P(Xt∈B(x,h))2| ≤g0(s−t)ϕ(h)2.
(H3) For any *t* ≥ 0, we set *ɛ*_*t*_∶ = Ψ(*Y*_*t*_) − *E*(Ψ(*Y*_*t*_)∣*X*_*t*_). There exists an integrable bounded function *g*_1_ on [0, *∞*) such that, for any (*s*, *t*)∈[0, *∞*)^2^, we have
  (5) max⁡{|E(ɛs ∣ Xs,Xt)|,|E(ɛsɛt ∣ Xs,Xt)|} ≤g1(|s−t|).
(H4) Let *𝒯*_*T*_ be the sigma-algebra generated by {*X*_*t*_, *t* ∈ [0, *T*]}. There exists a constant *R* > 0, not depending on *T*, such that
  (6) $$\begin{array}{c} \underset{t\in \left[0,T\right]}{\sup}E\left(\Psi {\left(Y_{t}\right)}^{2}\;|\;T_{T}\right)<R. \end{array}$$

## 3. Estimator and Result

We define the generalized regression function estimate by

(7) $$\begin{array}{c} {\hat{r}}_{T}\left(x\right)∶=\{\begin{array}{cc} \frac{\int_{t=0}^{T}\Psi \left(Y_{t}\right)K\left(h_{T}^{-1}d\left(x,X_{t}\right)\right)dt}{\int_{t=0}^{T}K\left(h_{T}^{-1}d\left(x,X_{t}\right)\right)dt} & \\ \;\text{if}\overset{T}{\underset{t=0}{\int }}K\left(h_{T}^{-1}d\left(x,X_{t}\right)\right)dt\neq 0, & \\ \frac{\int_{t=0}^{T}\Psi \left(Y_{t}\right)dt}{T}\;\text{otherwise}, & \end{array} \end{array}$$

where *K*(*x*) = *𝕀*_[0,1]_(*x*) is the indicator function on [0,1] and *h*_*T*_ is a bandwidth decreasing to 0 when *T* → *∞*. Remark that this estimator is the same as the one defined in [21, page 130] with the use of the semimetric *d* instead of the simple difference used in the real case.

Theorem 1 explores the performance of ${\hat{r}}_{T}(x)$ in terms of mean-square error.

**Theorem 1 .**

Suppose that (H1)–(H4) hold. Let *r* be (1) and ${\hat{r}}_{T}$ be (7) defined with *h*_*T*_ = *𝒪*(*T* ^−1/*η*^). Then, one has

(8) $$\begin{array}{c} \underset{x\in C}{\sup}\;E{\left({\hat{r}}_{T}\left(x\right)-r\left(x\right)\right)}^{2}=O\left(\frac{1}{T}\right). \end{array}$$

We can compare this rate of convergence with the one obtained for discrete time processes in [14], which is, with our notations,

(9) $$\begin{array}{c} {\left({\hat{r}}_{n}\left(x\right)-r\left(x\right)\right)}^{2}=O\left(h_{n}^{2}+\frac{1}{n\phi \left(h_{n}\right)}\right). \end{array}$$

Remark that, with infinite-dimensional variables, *ϕ*(*h*) can decrease to zero, when *h* tends to zero, at an exponential rate so that *h*_*n*_ have to tend to zero at a logarithmic rate.

## 4. Comments and Examples

(H1) is a very classical Hölderian condition on the true regression function, but, in the infinite-dimensional framework, this condition depends on the semimetric used.

The assumption on small balls probabilities given in (H2)-(i) is widely used in nonparametric estimation for functional data (see, e.g., the monograph [22]). However, we want to point out the fact that if we define equivalence classes using the semidistance *d*, we can construct a quotient space on which *d* is a distance and if this quotient space is infinite-dimensional, then this condition can be satisfied only very locally in that for any point *x* of our compact *𝒞*, we can find, for any *ɛ* > 0, a point *y* and a positive number *h* < *ɛ* such that *d*(*x*, *y*) < *ɛ* and *P*(*X*_0_ ∈ *ℬ*(*y*, *h*)) ≤ *β*_1_ *ϕ*(*h*): in that case, we could not extend our hypothesis to every point in an open ball (see [23] for a result on the consequences of a similar hypothesis on every point in a ball).

The most specific and restrictive assumption is (H2)-(ii), which is an adaptation to infinite-dimensional processes of the conditions on the density function introduced in [17] for real valued processes and transposed in [21, pages 135-136] to the estimation of the regression function with a vectorial regressor. Note that when *ℋ* = *ℝ* ^*d*^ and *ϕ*(*h*) = *h* ^*d*^, the rate of convergence obtained in Theorem 5.3 in [21, page 136] is the same as the one we obtain here, and the condition I2 used implies (H2)-(ii). On the other hand, processes can meet (H2)-(ii) and infringe the condition in [21], especially when the vectorial process *X*_*t*_ does not admit a density. For real valued processes, a slightly different version of the Castellana and Leadbetter hypothesis on the joint density is given in [24] where it is shown that this hypothesis is satisfied for a wide class of diffusion processes, including the Ornstein-Uhlenbeck Process: these processes are also examples of the range of applications of our result. Real continuous-time fractional ARMA processes studied in [25] are given as examples in [26]. Depending on the choice of the impulse response functions, a vector composed of such *d* processes can fulfil (H2)-(ii) for any *d*: using the notations of [25], if ((*X*_1,*T*_),…, (*X*_*d*,*T*_)) are *d* independent processes complying with conditions of Proposition 4 in [25] with *D* > −1/2 − 1/*d* and *a* > 0, then the vectorial process ((*X*_1,*T*_),…, (*X*_*d*,*T*_)) meets (H2)(ii). For processes valued in infinite-dimensional spaces, we can also give the example of hidden processes: let (*Z*_*t*_) be a nonobserved process valued in *ℝ* ^*d*^, for which conditions of Theorem 5.3 in [21, page 136] hold for every *x* in a compact *𝒜*, let Γ be an unknown function from *ℝ* ^*d*^ to a space *ℋ* (that can be infinite-dimensional) equipped with a semimetric *d*, and let (*X*_*t*_) = (Γ(*Z*_*t*_)) be an observed process. If there exist two positive constants (*a*, *b*) such that for any (*x*, *y*) ∈ *ℝ* ^*d*^ × *ℝ* ^*d*^,  *a*||*x* − *y*|| ≤ *d*(Γ(*x*), Γ(*y*)) ≤ *b*||*x* − *y*||, then (*X*_*t*_) fulfills (H2) with *ϕ*(*h*) = *h* ^*d*^ and *𝒞* = Ψ(*𝒜*). Note that even if *ℋ* = *ℝ* ^*d*′^ with *d*′ > *d*, (*X*_*t*_) does not satisfy the assumptions usually imposed to vectorial processes to obtain a superoptimal rate.

There are two conditions in (H3). The condition |*E*(*ɛ*_*s*_∣*X*_*s*_, *X*_*t*_)|≤*g*_1_(|*s* − *t*|) is less restrictive than imposing that the regressor and the noise are independent. |*E*(*ɛ*_*s*_ *ɛ*_*t*_∣*X*_*s*_, *X*_*t*_)|≤*g*_1_(|*s* − *t*|) is a weak condition on the decay of dependence as the distance between observations increases, and (*ɛ*_*t*_) may not be *α*-mixing. Note that we do not impose to (*ɛ*_*t*_) to be an irregular path process.

At last, it is much less restrictive to impose (H4) than to suppose that Ψ(*Y*_*t*_) is bounded. In particular, this assumption allows us to consider the model

(10) $$\begin{array}{c} \Psi \left(Y_{t}\right)=r\left(X_{t}\right)+{ɛ}_{t}, \end{array}$$

where *r* is a bounded function, (*ɛ*_*t*_) is a square integrable process, and *ɛ*_*t*_ and (*X*_*t*_) are independent.

On a given space, we can define many semidistances and hypotheses (H1)-(H2,) as well as the estimator itself, depending largely on the choice of this semidistance: the importance of this choice is widely discussed in [22] and a method to choose the semimetric for independent variables is proposed in [27], but this method does not ensure that (H1) holds. Actually, we can obtain a semimetric *d* such that *d*(*x*, *y*) = 0⇏*r*(*x*) = *r*(*y*). It would be of interest to develop a data driven method adapted to continuous time processes to select the semimetric.

In the statement of our theorem, we impose that *h*_*T*_ = *𝒯* ^−1/*η*^ where *η* is an unknown parameter so that the adaptation to continuous time processes of the method developed in [28] to choose the bandwidth would be interesting but is not in theory necessary in our framework. In point of fact, and it is what was very surprising when Castellana and Leadbetter first obtained a superoptimal rate of convergence, the bound for the variance of the estimator does not depend on *h*_*t*_ and we can choose *h*_*T*_ = *T* ^−log⁡(*T*)^ which will always satisfy *h*_*T*_ = *𝒯* ^−1/*η*^ for *T* large enough: even if this choice has no reason to be optimal, it leads to the claimed superoptimal rate of convergence.

Recently, results have been obtained in the case where the response *Y* is valued in a Banach space, which can be infinite-dimensional (see [29, 30]). Note that until Ψ is a real valued Borelian function, there is no need to change our proofs to obtain our result if *Y* is valued in a Banach space. However, in the case where Ψ(*Y*) is a Banach valued variable, we could not easily adapt our proofs and obtaining a superoptimal rate would involve very different techniques; it would be an interesting extension for further works.

## 5. Simulations

We chose *𝕃* ^2^([−1,1]) endowed with its natural norm as the functional space and simulated our process as follows.

At first we simulated an Ornstein-Uhlenbeck process solution of the stochastic differential equation

(11) $$\begin{array}{c} d{\text{OU}}_{t}=-9\left({\text{OU}}_{t}-2\right)dt+6dW_{t}, \end{array}$$

where *W*_*t*_ denotes a Wiener process. Here, we took *dt* = 0.0005.

Denoting the floor function by ⌊·⌋, let Γ be the function from *ℝ* to *𝕃* ^2^([−1,1]) defined by

(12) Γ(x)∶=(1+⌊x⌋−x)Pnum(⌊x⌋) +(x−⌊x⌋)Pnum(⌊x+1⌋) ∀x∈R,

where *P*_*i*_ is the Legendre polynomial of degree *i* and num(*x*)∶ = 1 + 2 × sign⁡(*x*) × *x* − sign⁡(*x*)×(1 + sign⁡(*x*))/2. Then we define our functional process for any *t* ∈ [0, *T*] setting

(13) $$\begin{array}{c} X_{t}∶=\Gamma \left({\text{OU}}_{t}\right). \end{array}$$

For any square integrable function *x* on [−1,1], we define the function

(14) $$\begin{array}{c} r\left(x\right)∶=\overset{1}{\underset{u=-1}{\int }}x\left(u\right)\left(2u+x\left(u\right)\right)du \end{array}$$

and set

(15) $$\begin{array}{c} Y_{t}=r\left(X_{t}\right)+U_{t}, \end{array}$$

where *U*_*t*_ = *W*_*t*_′ − *W*_*T*−1_′ and *W*_*t*_′ is a Wiener process independent of *X*.

**Figure 1:**
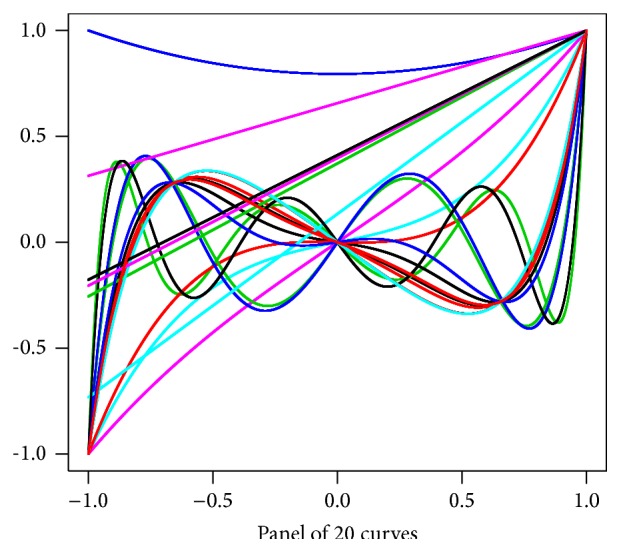


In order to obtain a panel of 20 points (in *𝕃* ^2^([−1,1])) where we can evaluate the regression function, we did a first simulation with *T* = 10 and set *𝒞*∶ = (*X*_*i*/2_, *i* ∈ 1,2,…, 20). Once obtained, *𝒞* is considered as a deterministic set. We represent these functions in Figure 1.

*Remark. *We check here that the simulated processes fulfil our hypotheses.

At first, denoting by Id the identity function on [−1,1], for any (*x*, *y*) ∈ *𝕃* ^2^([−1,1]) × *𝕃* ^2^([−1,1]), we have

(16) |r(x)−r(y)|=|||x||2−||y||2+2〈x−y,Id〉|≤(||x+y||+2)||x−y||

and *r* satisfies (H1) with *η* = 1.

The Ornstein-Uhlenbeck process satisfies the part of Condition I2 on the regressor's density in [21, page 136]. Moreover, Γ is a bijection from *ℝ* to *Im*⁡(Γ), and it can be shown that, for some constant *C*, there exist 0 < *a* < *b* such that for any 0 < *h* < *C* and any *x* ∈ Γ^−1^(*𝒞*), the two following implications are correct:

(17) (z∈B(x,ah))⟹(Γ(z)∈B(Γ(x),h)),(Γ(z)∈B(x,h))⟹(z∈B(x,bh)),

which implies that (H2)(i)-(ii) are fulfilled when taking *ϕ*(*h*) = *h*.

Since (*ɛ*_*t*_) and (*X*_*t*_) are independent and Cov(*ɛ*_*t*_, *ɛ*_*s*_) = 0 if |*t* − *s* | >1, (H3) is satisfied.

Finally, the model used in the simulation corresponds to the choice of the identity function for Ψ in (1), where (*Y*_*t*_) is an unbounded process and *r*(·) is not a bounded function. However, *r*(·) is bounded on *Im*⁡(Γ) and so (H4) is fulfilled.

**Figure 2:**
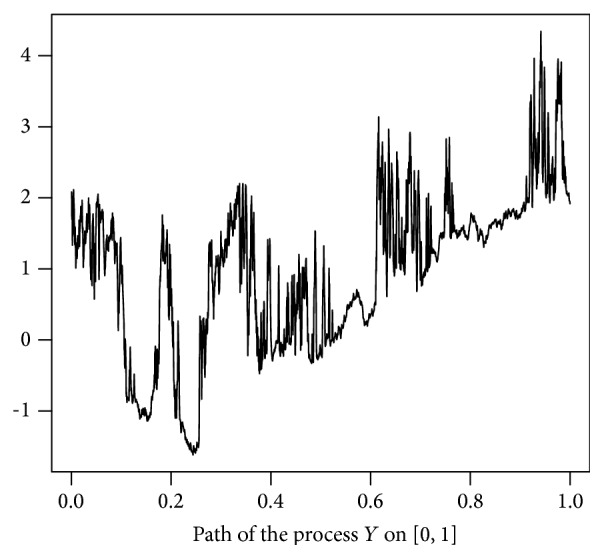


We simulated the paths of the process (*X*_*t*_, *Y*_*t*_)_*t*∈[0,*T*]_ for different values of *T*.  Figure 2 represents the path of the process (*Y*_*t*_) for *t* ∈ [0,1].

We estimated the regression function at each point in *𝒞*, for different values of *T*, and compared our results to those obtained when studying a discrete time functional process, that is, when we observe (*X*_*t*_, *Y*_*t*_) only for *t* ∈ *ℕ*, and we use the estimator defined in [12] with the indicator function as the kernel: it corresponds to an infinite-dimensional version of Nadaraya-Watson estimator with a uniform kernel. When working with the discrete time process we used the data-driven way of choosing the bandwidth proposed in [28]. When working with the continuous time process that is observed on a very thin grid, for *T* = 50, we chose the same bandwidth as the one used for the discrete time process and, for *T* > 50, we supposed *r* to be Lipschitz (i.e., *η* = 1, which is the case here) and used the bandwidth *h*_*T*_ = *h*_50_(50/*T*). In Table 1, we give the mean square error evaluated on the functions of the panel for different *T* = 50, 500, and 2000.

We can see that, for *T* = 50, we already have a smaller mean square error with the estimator using the continuous time process, and when *T* increase, the mean square error seems to decrease much more quickly when working with the continuous time process. However, the continuous time approach takes much more time and much more memory; we had to split the calculation into several parts and delete intermediate calculations to avoid saturating memory.

**Figure 3:**
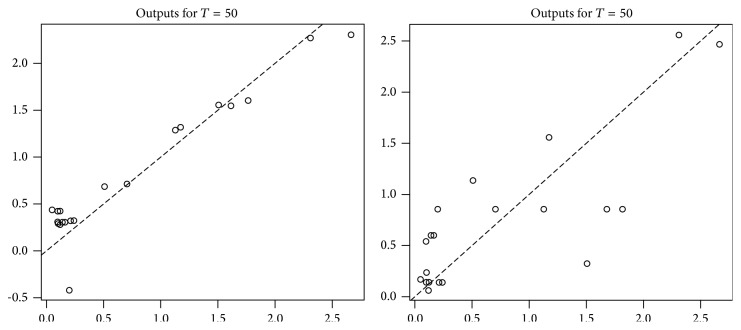
Continuous time estimator (left) and discrete time estimator (right); in abscissa the value of the real regression function applied to each function of our panel and in ordinate the estimated value of the regression function.

**Figure 4:**
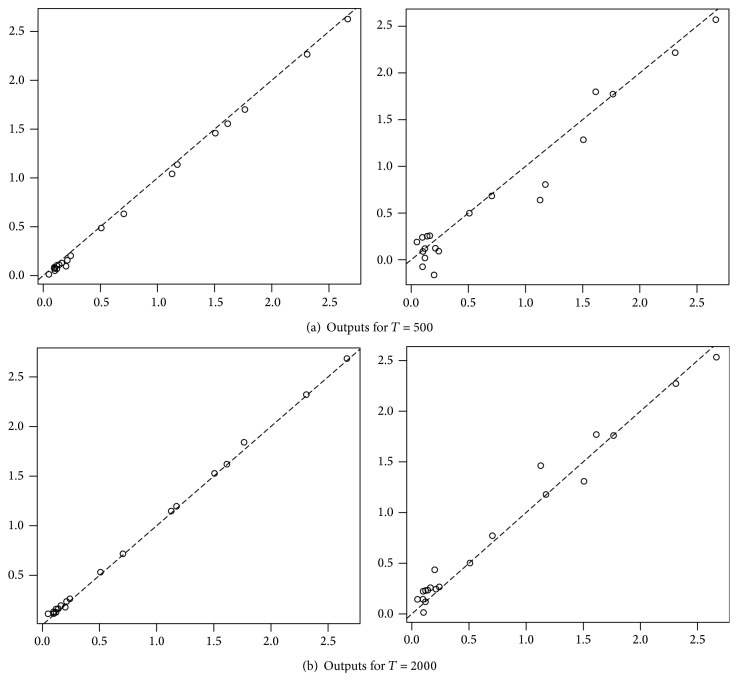
Continuous time estimator (left) and discrete time estimator (right); in abscissa the value of the real regression function applied to each function of our panel and in ordinate the estimated value of the regression function.

In Figures 3 and 4, we have in abscissa the value of the real regression function applied to each function of our panel and in ordinate the estimated value of the regression function. We represent on the left the results for the continuous time estimator and on the right the results for the discrete time estimator.

## 6. Proofs

### 6.1. Intermediary Results

In the sequel, we use the following notations:

(18) $$\begin{array}{c} \Delta_{T,t}\left(x\right)=K\left(h_{T}^{-1}d\left(x,X_{t}\right)\right),\\ {\hat{r}}_{1,T}\left(x\right)∶=\frac{1}{TE\left(\Delta_{T,0}\left(x\right)\right)}\overset{T}{\underset{t=0}{\int }}\Delta_{T,t}\left(x\right)dt,\\ {\hat{r}}_{2,T}\left(x\right)∶=\frac{1}{TE\left(\Delta_{T,0}\left(x\right)\right)}\overset{T}{\underset{t=0}{\int }}\Psi \left(Y_{t}\right)\Delta_{T,t}\left(x\right)dt. \end{array}$$

Lemma 2 below studies the behavior of the bias of ${\hat{r}}_{2,T}$.

**Lemma 2 .**

Under the conditions of Theorem 1, one has

(19) $$\begin{array}{c} \underset{x\in C}{\sup}|E\left({\hat{r}}_{2,T}\left(x\right)\right)-r\left(x\right)|=O\left(\frac{1}{T}\right). \end{array}$$

Lemma 3 below provides an upper bound for the variances of ${\hat{r}}_{1,T}$ and ${\hat{r}}_{2,T}$.

**Lemma 3 .**

Under the conditions of Theorem 1, one has

(20) $$\begin{array}{c} \underset{x\in C}{\sup}\left(\mathrm{Var}\left({\hat{r}}_{2,T}\left(x\right)\right)+\mathrm{Var}\left({\hat{r}}_{1,T}\left(x\right)\right)\right)=O\left(\frac{1}{T}\right). \end{array}$$

### 6.2. Proofs of the Intermediary Results

For the sake of conciseness, when no confusion is possible, we use the notations Ψ_*t*_∶ = Ψ(*Y*_*t*_) and Δ_*T*,*t*_∶ = Δ_*T*,*t*_(*x*).

**Proof of Lemma 2.**

Observe that, for any *x* ∈ *𝒞*,

(21) E(r^2,T(x))=1TE(ΔT,0)∫t=0TE(ΨtΔT,t)dt=E(Ψ0ΔT,0)E(ΔT,0)=E(E(r(X0)+ɛ0 ∣ X0)ΔT,0)E(ΔT,0)=E(r(X0)ΔT,0)E(ΔT,0).

Hence,

(22) E(r^2,T(x))−r(x)=E(r(X0)ΔT,0)E(ΔT,0)−r(x)=E((r(X0)−r(x))ΔT,0)E(ΔT,0).

Owing to (H1), we have |*r*(*X*_0_) − *r*(*x*)|Δ_*T*,0_ ≤ Δ_*T*,0_ sup⁡_*u*∈*ℬ*(*x*,*h*_*T*_)_|*r*(*u*) − *r*(*x*)| ≤ *C*Δ_*T*,0_ *h*_*T*_^*η*^. Therefore, by Jensen's inequality and *h* ^*η*^ = *𝒪*(1/*T*), we have

(23) sup⁡x∈C|E(r^2,T(x))−r(x)| ≤sup⁡x∈CE(|r(X0)−r(x)|ΔT,0)E(ΔT,0)≤ChTη=O(1T).

This ends the proof of Lemma 2.

**Proof of Lemma 3.**

For any *x* ∈ *𝒞*, by Fubini's Theorem, we have

(24) Var⁡(r^2,T(x)) =1T2E(ΔT,0)2∫t=0T∫s=0TCov(ΨsΔT,s,ΨtΔT,t)dt ds.

*Upper Bound of the Covariance Term.* In order to simplify the notations, we set *R*(*X*_*t*_)∶ = *E*(Ψ_*t*_∣*X*_*t*_) and *ɛ*_*t*_∶ = Ψ_*t*_ − *R*_*t*_. Note that

(25) E(ΨsΨt ∣ Xs,Xt)=R(Xs)R(Xt)+R(Xs)E(ɛt ∣ Xs,Xt) +R(Xt)E(ɛs ∣ Xs,Xt) +E(ɛsɛt ∣ Xs,Xt).

Therefore, the covariance term can be expended as follows:

(26) Cov(ΨsΔT,s,ΨtΔT,t)=E(ΨsΔT,sΨtΔT,t) −E(ΨsΔT,s)E(ΨtΔT,t)=E(ΔT,sΔT,tE(ΨsΨt ∣ Xs,Xt)) −E(ΔT,sR(Xs))2=E(ΔT,sΔT,tR(Xs)R(Xt)) +E(ΔT,sΔT,t(R(Xs)E(ɛt ∣ Xs,Xt)iiiiiiiiiiiiiiiiiiiiiii+R(Xt)iiiiiiiiiiiiiiiiiiiiiii× E(ɛs ∣ Xs,Xt))) +E(ΔT,sΔT,tE(ɛsɛt ∣ Xs,Xt)) −E(ΔT,sR(Xs))2.

Set

(27) $$\begin{array}{c} d_{t}∶=R\left(X_{t}\right)-r\left(x\right). \end{array}$$

We have

(28) Cov(ΨsΔT,s,ΨtΔT,t)=r(x)2E(ΔT,sΔT,t) +r(x)(E(ΔT,sΔT,tdt)hhhhihiiih+E(ΔT,sΔT,tds)) +E(ΔT,sΔT,tdtds)+r(x) ×E(ΔT,sΔT,t(E(ɛt ∣ Xs,Xt)hhhhhhihhhihhh+E(ɛs ∣ Xs,Xt))) +E(ΔT,sΔT,thhhiihh×(dsE(ɛt ∣ Xs,Xt)hhhhhiihii+dtE(ɛs ∣ Xs,Xt))) +E(ΔT,sΔT,tE(ɛsɛt ∣ Xs,Xt)) −r(x)2E(ΔT,s)2−E(ΔT,sds)2 −2r(x)E(ΔT,sds)E(ΔT,s)=r(x)2(E(ΔT,sΔT,t)−E(ΔT,s)2) −(E(ΔT,sds)2hhhiihh+2r(x)E(ΔT,sds)E(ΔT,s)) +E(ΔT,sΔT,tQ),

with

(29) Q=dsE(ɛt ∣ Xs,Xt)+dtE(ɛs ∣ Xs,Xt) +dsdt+E(ɛsɛt ∣ Xs,Xt) +r(x)(ds+dt+E(ɛt ∣ Xs,Xt)+E(ɛs ∣ Xs,Xt)).

The triangular inequality and Jensen's inequality yield

(30) $$\begin{array}{c} \left|\text{Cov}\left(\Psi_{s}\Delta_{T,s},\Psi_{t}\Delta_{T,t}\right)\right|\leq L+M+N, \end{array}$$

where

(31) $$\begin{array}{c} L=r{\left(x\right)}^{2}\left|E\left(\Delta_{T,s}\Delta_{T,t}\right)-E{\left(\Delta_{T,s}\right)}^{2}\right|,\\ M=E{\left(\Delta_{T,s}\left|d_{s}\right|\right)}^{2}+2\left|r\left(x\right)\right|E\left(\Delta_{T,s}\left|d_{s}\right|\right)E\left(\Delta_{T,s}\right),\\ N=E\left(\Delta_{T,s}\Delta_{T,t}\left|Q\right|\right). \end{array}$$

*Upper Bound for L*. Using (H2)-(ii), we have

(32) $$\begin{array}{c} L\leq r{\left(x\right)}^{2}g_{0}\left(\left|s-t\right|\right)\phi {\left(h_{T}\right)}^{2}. \end{array}$$

*Upper Bound for M*. Owing to (H1), we have Δ_*T*,*s*_|*d*_*s*_| ≤ Δ_*T*,*s*_sup⁡_*u*∈*ℬ*(*x*,*h*_*T*_)_|*r*(*u*) − *r*(*x*)| ≤ *C*Δ_*T*,*s*_ *h*_*T*_^*η*^. It follows from this inequality and (H2)-(i) that

(33) M≤(2|r(x)|ChTη+C2hT2η)E(ΔT,s)2≤(2r(x)ChTη+C2hT2η)β22ϕ(hT)2.

*Upper Bound for N*. By similar techniques to those in the bound for *M* and (H3), we obtain

(34) ΔT,sΔT,t|Q|≤ΔT,sΔT,t ×(2|r(x)|ChTη+C2hT2ηiiiiiiii+(2(|r(x)|+ChTη)+1)g1(|s−t|)(2|r(x)|ChTη+C2hT2η).

On the other hand, by (H2)-(ii),

(35) E(ΔT,sΔT,t)≤|Cov(ΔT,s,ΔT,t)|+E(ΔT,s)2≤(β22+g0(|s−t|))ϕ(hT)2.

Hence,

(36) N≤(2|r(x)|ChTη+C2hT2ηiiiii+(2(|r(x)|+ChTη)+1)g1(|s−t|)(2|r(x)|ChTη+C2hT2η) ×(β22+g0(|s−t|))ϕ(hT)2.

Therefore, setting

(37) GT(y)∶=r(x)2g0(y)ϕ(hT)2 +(2|r(x)|ChTη+C2hT2ηiiiiiiii+(2(|r(x)|+ChTη)+1)g1(y)(2|r(x)|ChTη+C2hT2η) ×(β22+g0(y))ϕ(hT)2 +(2r(x)ChTη+C2hT2η)β22ϕ(hT)2,

the obtained upper bounds for *L*, *M*, and *N* yield

(38) |Cov(ΨsΔT,s,ΨtΔT,t)| ≤GT(|s−t|).

*Final Bound*. Combining (24) and (38) and using (H2)-(i), we have

(39) Var⁡(r^2,T(x))≤2T2E(ΔT,0)2∫t=0T∫s=tTGT(s−t)dt ds≤2TE(ΔT,0)2∫y=0TGT(y)dy≤2Tβ12ϕ(hT)2∫y=0TGT(y)dy.

Since *g*_0_ and *g*_1_ are integrable and *r* is bounded on *𝒞* and *h* ^*η*^ = *𝒪*(1/*T*), there exists a constant *C*_0_ such that

(40) $$\begin{array}{c} \underset{x\in C}{\sup}\mathrm{Var}\left({\hat{r}}_{2,T}\left(x\right)\right)\leq \frac{C_{0}}{T}. \end{array}$$

The special choice of Ψ : (*x*) ↦ 1 leads us to

(41) $$\begin{array}{c} \underset{x\in C}{\sup}\mathrm{Var}\left({\hat{r}}_{1,T}\left(x\right)\right)\leq \frac{C_{1}}{T}. \end{array}$$

This last inequality concludes the proof of Lemma 3.

**Proof of Theorem 1.**

We can write

(42) r^T(x)−r(x)=(r^T(x)−r^2,T(x)) +(r^2,T(x)−E(r^2,T(x))) +(E(r^2,T(x))−r(x)).

The elementary inequality: (*a*+*b*+*c*)^2^ ≤ 3(*a* ^2^ + *b* ^2^ + *c* ^2^), (*a*, *b*, *c*) ∈ (0,*∞*)^3^, yields

(43) $$\begin{array}{c} \underset{x\in C}{\sup}\;E{\left({\hat{r}}_{T}\left(x\right)-r\left(x\right)\right)}^{2}\leq 3\left(U+V+W\right), \end{array}$$

where

(44) $$\begin{array}{c} U=\underset{x\in C}{\sup}\;E{\left({\hat{r}}_{T}\left(x\right)-{\hat{r}}_{2,T}\left(x\right)\right)}^{2},\\ V=\underset{x\in C}{\sup}\;E{\left({\hat{r}}_{2,T}\left(x\right)-E\left({\hat{r}}_{2,T}\left(x\right)\right)\right)}^{2},\\ W=\underset{x\in C}{\sup}{\left(E\left({\hat{r}}_{2,T}\left(x\right)\right)-r\left(x\right)\right)}^{2}. \end{array}$$

*Upper Bound for V*. Lemma 3 yields

(45) $$\begin{array}{c} V=\underset{x\in C}{\sup}\mathrm{Var}\left({\hat{r}}_{2,T}\left(x\right)\right)=O\left(\frac{1}{T}\right). \end{array}$$

*Upper Bound for W*. Lemma 2 yields

(46) $$\begin{array}{c} W=O\left(\frac{1}{T^{2}}\right). \end{array}$$

*Upper Bound for U*. We define, for any *t* ∈ [0, *T*], the quantity:

(47) $$\begin{array}{c} Z_{t}∶=\{\begin{array}{cc} \frac{K\left(h_{T}^{-1}d\left(x,X_{t}\right)\right)}{\int_{t=0}^{T}K\left(h_{T}^{-1}d\left(x,X_{t}\right)\right)dt} & \\ \;\text{if}\;\overset{T}{\underset{t=0}{\int }}K\left(h_{T}^{-1}d\left(x,X_{t}\right)\right)dt\neq 0, & \\ \frac{1}{T}\;\text{otherwise}. & \end{array} \end{array}$$

Note that, when ${\hat{r}}_{1,T}\left(x\right)\neq 0$,

(48) $$\begin{array}{c} {\hat{r}}_{2,T}\left(x\right)={\hat{r}}_{T}\left(x\right)\times {\hat{r}}_{1,T}\left(x\right), \end{array}$$

so that

(49) U≤sup⁡x∈C E(r^T(x)(1−r^1,T(x)))2 +E(I{0}(r^1,T(x))∫0TΨ(Yt)Tdt)  .

Using (H4) and Lemma 3, we get

(50) sup⁡x∈C E(r^T(x)(1−r^1,T(x)))2 =sup⁡x∈C E(E(r^T2(x)(1−r^1,T(x))2 ∣ TT)) =sup⁡x∈C E(E(r^T2(x) ∣ TT)iiiiiiiiiiiiiiiiii×(1−r^1,T(x))2) =sup⁡x∈C E(E((∫t=0TZtΨ(Yt)dt)2 ∣ TT)iiiiiiiiiiiiiiiiiiiiiii× (1−r^1,T(x))2((∫t=0TZtΨ(Yt)dt)2 ∣ TT)) =sup⁡x∈C E(∫(s,t)∈[0,T]2ZtZsE(Ψ(Yt)Ψ(Ys) ∣ TT)ds dtiiiiiiiiiiiiiiiii× (1−r^1,T(x))2∫(s,t)∈[0,T]2) ≤sup⁡x∈C E(∫(s,t)∈[0,T]2ZtZsR ds dtiiiiiiiiiiiiiiiii×(1−r^1,T(x))2∫(s,t)∈[0,T]2) ≤sup⁡x∈C RE((1−r^1,T(x))2) =sup⁡x∈C RVar⁡(r^1,T(x))=O(1T).

Similarly, (H4), Lemma 3, and Chebyshev's inequality lead to

(51) sup⁡x∈C E(I{0}(r^1,T(x))∫0TΨ(Yt)Tdt) ≤sup⁡x∈C RE(I{0}(r^1,T(x))) ≤sup⁡x∈CRP(|r^1,T(x)−1|≥1) ≤RT.

We finally obtain

(52) $$\begin{array}{c} U=O\left(\frac{1}{T}\right). \end{array}$$

Putting the obtained upper bounds for *U*, *V*, and *W* together, we have

(53) $$\begin{array}{c} \underset{x\in C}{\sup}\;E{\left({\hat{r}}_{T}\left(x\right)-r\left(x\right)\right)}^{2}=O\left(\frac{1}{T}\right). \end{array}$$

Theorem 1 is proved.

## Figures and Tables

**Table 1:** 

	Continuous time process	Discrete time process
*T* = 50	0.056623	0.231032
*T* = 500	0.003235	0.037855
*T* = 2000	0.000698	0.0155137
